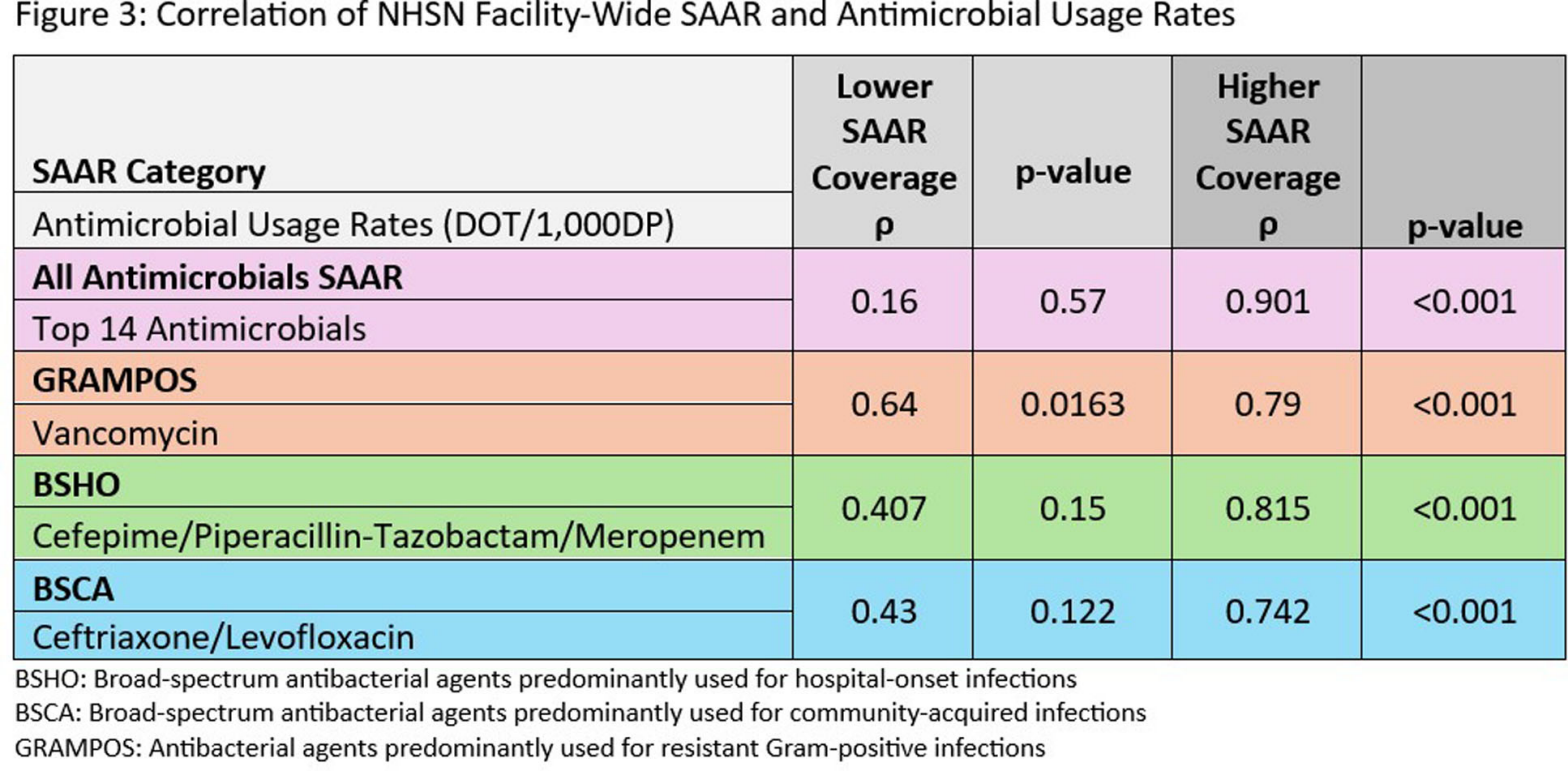# 63 Persistence of Legionella in Healthcare Ice Machines Compared with Other Water Sources

**DOI:** 10.1017/ash.2026.10494

**Published:** 2026-06-23

**Authors:** Jenna Preusker, M. Salman Ashraf, Trevor Van Schooneveld, Scott Bergman, Danny Schroeder, Rabia Syed, Lacey Pavlovsky, Juan Teran Plasencia

**Affiliations:** 1 Nebraska Medicine; 2 University of Nebraska Medical Center; 3 Nebraska Medicine / Nebraska ASAP; 4 California Department of Public Health

## Abstract

**Background:** Antimicrobial stewardship programs use the NHSN standardized antimicrobial administration ratio (SAAR) to monitor antimicrobial use (AU) and direct activities, but only certain locations generate SAAR values. We explored the correlation between hospital SAAR coverage using days present (DP) and facility-wide AU measured in days of therapy per 1,000 days present (DOT/1,000DP). **Methods:** We included Nebraska hospitals reporting at least one adult location and at least six months of AU data that generated a SAAR during 7/2024-6/2025. We classified hospitals by bed size [large: <150, medium: 26–150, and critical access hospitals: ≤25]. Days present from SAAR eligible locations were divided by total facility DP to calculate the SAAR coverage proportion. We categorized hospitals in the upper third as having high SAAR coverage and those in the lower two-thirds as low SAAR coverage. We calculated Spearman correlation coefficients to evaluate the strength of the association between SAAR and AU rates in the high and low SAAR coverage groups. **Results:** Overall, 45 hospitals were included (median 25 beds, range 7-641) with large variability among total days present (473-228,051). Regarding SAAR coverage, 14 hospitals were classified as low coverage and 31 had high coverage (representing 47% and 83% of total DP, respectively), with an overall median SAAR coverage of 91% (Figure 1). When comparing the all-antimicrobial SAAR with the total AU rate of the top 14 antimicrobials, hospitals with low SAAR coverage showed weak correlation (? 0.163, p-value=0.57) compared to those with high coverage (? 0.901, p-value=<0.001) (Figure 2). Performance varied for other NHSN SAAR categories and AU rates in the low coverage group, with higher correlation between the GRAMPOS SAAR category and vancomycin DOT/1,000DP, and lower correlation between the BSHO SAAR category and the total AU rate of cefepime/piperacillin-tazobactam/meropenem use (Figure 3). **Conclusion:** In hospitals with low SAAR coverage, SAAR values may not accurately reflect overall facility-wide AU. This discrepancy is especially evident when evaluating overall antimicrobial use. We hypothesize that the weak correlation is likely a result of substantial AU in locations that do not generate a SAAR. We noted variation in AU rates in hospitals with similar SAAR values which may stem from the CDC’s predictive model although incomplete capture of antimicrobial use may be playing some role as well. These findings support CDC efforts to expand the SAAR to include new locations.

**Figure:**
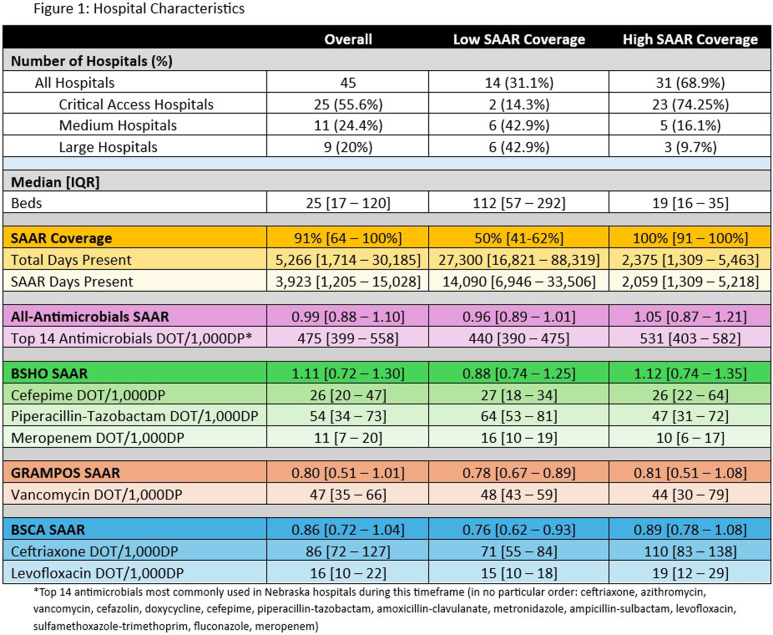

**Figure:**
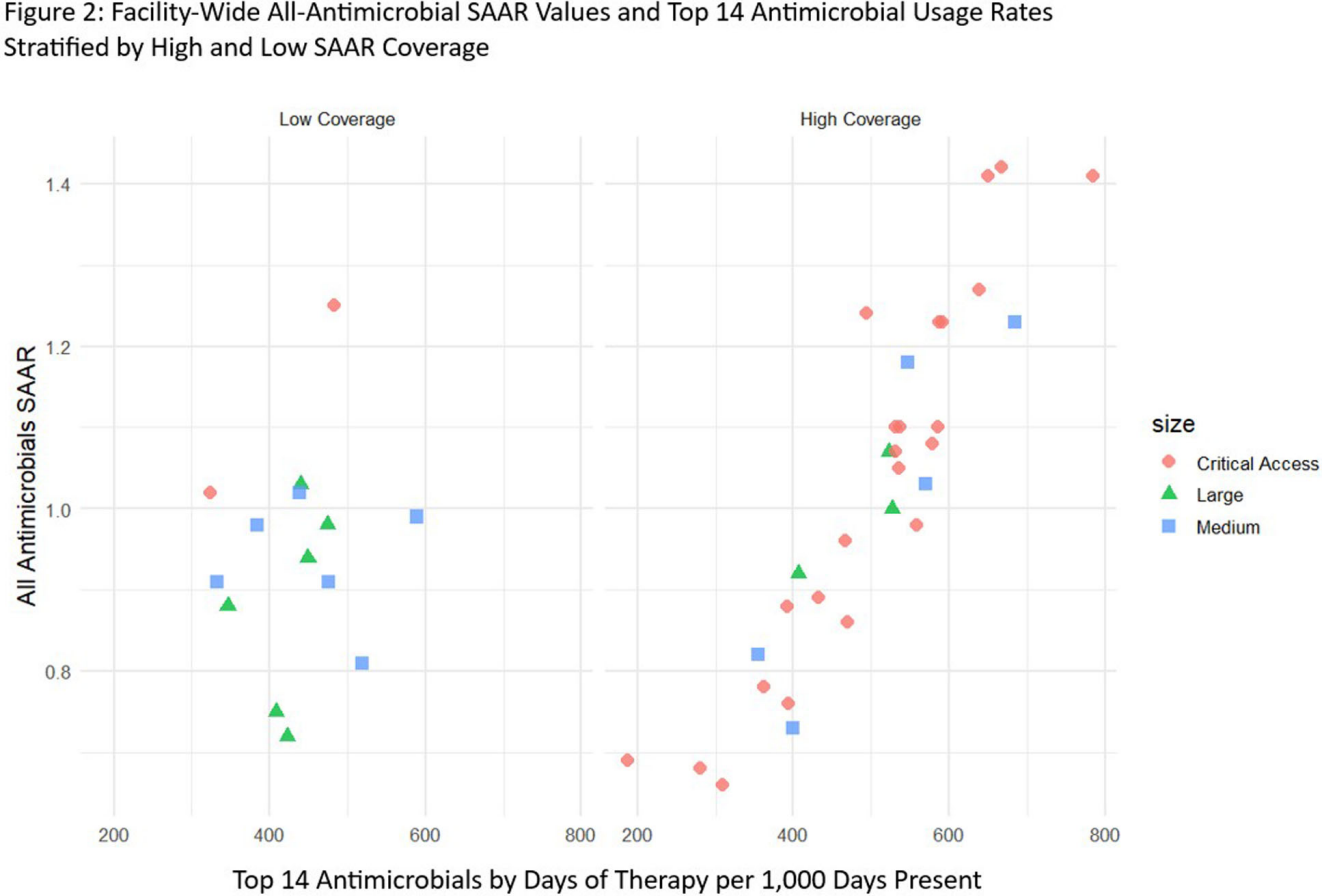

**Figure:**